# Business Return in New Orleans: Decision Making Amid Post-Katrina Uncertainty

**DOI:** 10.1371/journal.pone.0006765

**Published:** 2009-08-26

**Authors:** Nina S. N. Lam, Kelley Pace, Richard Campanella, James LeSage, Helbert Arenas

**Affiliations:** 1 Department of Environmental Sciences, Louisiana State University, Baton Rouge, Louisiana, United States of America; 2 Department of Finance, Louisiana State University, Baton Rouge, Louisiana, United States of America; 3 Center for Bioenvironmental Research, Tulane University, New Orleans, Louisiana, United States of America; 4 Department of Finance and Economics, Texas State University, San Marcos, Texas, United States of America; University of East Piedmont, Italy

## Abstract

**Background:**

Empirical observations on how businesses respond after a major catastrophe are rare, especially for a catastrophe as great as Hurricane Katrina, which hit New Orleans, Louisiana on August 29, 2005. We analyzed repeated telephone surveys of New Orleans businesses conducted in December 2005, June 2006, and October 2007 to understand factors that influenced decisions to re-open amid post-disaster uncertainty.

**Methodology/Principal Findings:**

Businesses in the group of professional, scientific, and technical services reopened the fastest in the near term, but differences in the rate of reopening for businesses stratified by type became indistinguishable in the longer term (around two years later). A reopening rate of 65% was found for all businesses by October 2007. Discriminant analysis showed significant differences in responses reflecting their attitudes about important factors between businesses that reopened and those that did not. Businesses that remained closed at the time of our third survey (two years after Katrina) ranked levee protection as the top concern immediately after Katrina, but damage to their premises and financing became major concerns in subsequent months reflected in the later surveys. For businesses that had opened (at the time of our third survey), infrastructure protection including levee, utility, and communications were the main concerns mentioned in surveys up to the third survey, when the issue of crime became their top concern.

**Conclusions/Significance:**

These findings underscore the need to have public policy and emergency plans in place prior to the actual disaster, such as infrastructure protection, so that the policy can be applied in a timely manner before business decisions to return or close are made. Our survey results, which include responses from both open and closed businesses, overcome the “survivorship bias” problem and provide empirical observations that should be useful to improve micro-level spatial economic modeling of factors that influence business return decisions.

## Introduction

On August 29, 2005, a surge of Gulf of Mexico water induced by Hurricane Katrina's high winds and low barometric pressure penetrated a network of manmade navigation and drainage canals in the sea-level-straddling metropolis of New Orleans, Louisiana. Some levees along those canals were overtopped and disintegrated; others, heightened with concrete floodwalls, deteriorated from below. The multiple catastrophic failures sent high-velocity torrents of salt water into certain neighborhoods and spilled into adjacent hydrological sub-basins. By mid-week, seawater filled nearly every basin east of the Mississippi River in New Orleans proper, plus all those in neighboring St. Bernard and one in Jefferson parishes. Above-sea-level neighborhoods either evaded the deluge or saw one to three feet of water; below-sea-level areas, which comprise half of the metropolis, suffered six to twelve feet. Floodwaters covered roughly four-fifths of the urbanized footprint of New Orleans on the East Bank. Close to two-thirds of New Orleanians had their residences inundated. Over five hundred people perished during the storm, followed a roughly equal number during the chaotic one-week aftermath, plus another five hundred during the traumatic months of evacuation that lay ahead. Katrina's death toll in the New Orleans area totaled over 1600, not including over two hundred who perished in nearby coastal Mississippi. By early September, vast expanses of Orleans, St. Bernard, and Plaquemines parishes were without population and economic activity [1].

A few thousand evacuees began returning in late September, to reoccupy their homes or assess the wreckage. By early October, scores of businesses had reopened, mostly in unflooded middle-class neighborhoods. Seventy-five percent of the hundreds of businesses on dry, prosperous Magazine Street, for example, reopened within three months of the catastrophe, while only seven percent of those along lightly flooded, poor St. Claude Avenue reopened in the same period. Heavily flooded areas—particularly poor ones—remained entirely without commerce. By the new year, about 144,000 residents out of the pre-Katrina population of approximately 450,000 occupied their homes, while owners of businesses pondered how to respond to the dynamic new environment. They did so against a backdrop of great uncertainty. In New Orleans alone, over 107,000 housing units were damaged by flooding (most severely), while another 27,000 suffered wind damage. Electricity, gas, and potable water—critical for both residential as well as commercial activity—returned piecemeal to unflooded areas throughout mid-autumn, but did not reach flooded regions until well into 2006 and remained tenuous citywide into 2007 [2].

During this time, citizens engaged in a series of urban planning efforts, overseen by such entities as the Urban Land Institute, the Bring New Orleans Back Commission, the Unified New Orleans Plan (UNOP), and others, One key question at each forum entailed whether the entire city would be rebuilt, or whether low-lying, far-flung subdivisions should be converted to green space. “Shrinking the city's footprint” became a political volatile issue, which coincided with a nationally watched mayoral campaign. Planners generally supported the concept, while social activists resisted it fiercely and sometimes equated it with ethnic and class cleansing. This resistance, coupled with the legal and financial difficulty of closing down neighborhoods and compensating homeowners, politically doomed the effort to shrink the urban footprint. Mayor C. Ray Nagin (who cinched re-election in May 2006) generally held a *laissez-faire* repopulation and rebuilding stance, saying, in effect, *let people return and rebuild as they can and as they wish, and we'll act on the patterns as they fall in place*. Federal complicity played a role as well: FEMA's updated Advisory Base Flood Elevation maps—which drive flood insurance availability and rates—turned out to be largely the same as the old 1984 maps, thus seemingly communicating federal endorsement (as well as actuarial encouragement) to homeowners deliberating on whether to rebuild in low-lying areas. Funds from the Louisiana Road Home—the state program compensating flooded homeowners with $150,000 minus insurance settlements and FEMA grants—imparted no special incentive to do otherwise, and no federal compensation fund awaited those homeowners and businesses that would have been affected by a hypothetical footprint-shrinkage decision. The decision that the entire city would be rebuilt gave a green light to business owners in heavily damaged neighborhoods to continue considering reopening in place, but by no means guaranteed their safety or success [1].

Citizens looked to the U.S. Army Corps of Engineers, the department responsible for the levee failures, for estimating their level of risk in face of future storms and rising sea levels. By 2007, the Corps allocated $15 billion toward protecting the city against the threat presented by a storm with a one-percent chance of occurring in any given year (the so-called “hundred-year storm”). The effort, scheduled for completion in 2011, involves the strengthening of certain levees, the building of flood gates on drainage and navigation canals, and the installation of pumps to remove runoff within the city's basins. The 2011 promise falls well short of protecting the city, serving only to reduce its risk. That the 2006–2007 storm seasons proved to be mild and event-free, especially compared to the extremely busy and deadly 2005 season, probably factored into many business reopening decisions [1].

Within city limits, social changes also affected those decisions. The low crime rate of the first post-Katrina autumn did not last; for reasons that continue to be debated, violent crime soared throughout 2006, climaxing (in perception if not in reality) with a march of thousands of citizens upon City Hall in January 2007, following two high-profile murders. The presence of National Guard troops patrolling the streets of an American city for years after the storm imparted a disquieting image. Crime, coupled with a deeply troubled parish-led public education system that was largely supplanted after the storm by state management or charterization, made New Orleans an unattractive option to many families considering returning here or relocating here. Insurance controversies, ill-run city services, and bleak news from scientists regarding rising seas and sinking soils, conspired further to complicate decision-making processes. This study seeks to understand how business owners in post-Katrina New Orleans negotiated that process.

Businesses do not make such decisions in a vacuum. In a complex human social dynamic system, decision makers in a post-catastrophic event include residents, businesses, and policy makers. While there is a rich literature on proposed theoretical models of decision making under uncertainty [3], [4], decision making related to catastrophic events is not well understood [5]. These events are rare, exhibit high levels of uncertainty, and offer few objective sources of information, limiting opportunities for systematic study based on empirical observations and formal methods of statistical inference. This is especially true regarding business decisions after a major catastrophe such as Hurricane Katrina. In general, we know more about how individuals make decisions in situations involving risk [6]–[8], but very little about decisions by businesses, groups, and organizations, which have large direct as well as indirect or spatial spillover impacts on the community at large. Moreover, literature directly addressing the relationship between business recovery and natural disasters remains sparse and mixed [9]–[14]. A better understanding of factors influencing business decisions to return after a major disaster requires fine-scale empirical observations collected in a timely manner. This type of information allows analysis of spatiotemporal changes in factors that influence decision making as well as interdependence between decisions made by one establishment and its neighbors. The ultimate goal of fine-scale analyses is to derive empirical rules so that quantitative models can be developed to understand and predict business return after a major disaster.

**Figure 1:**
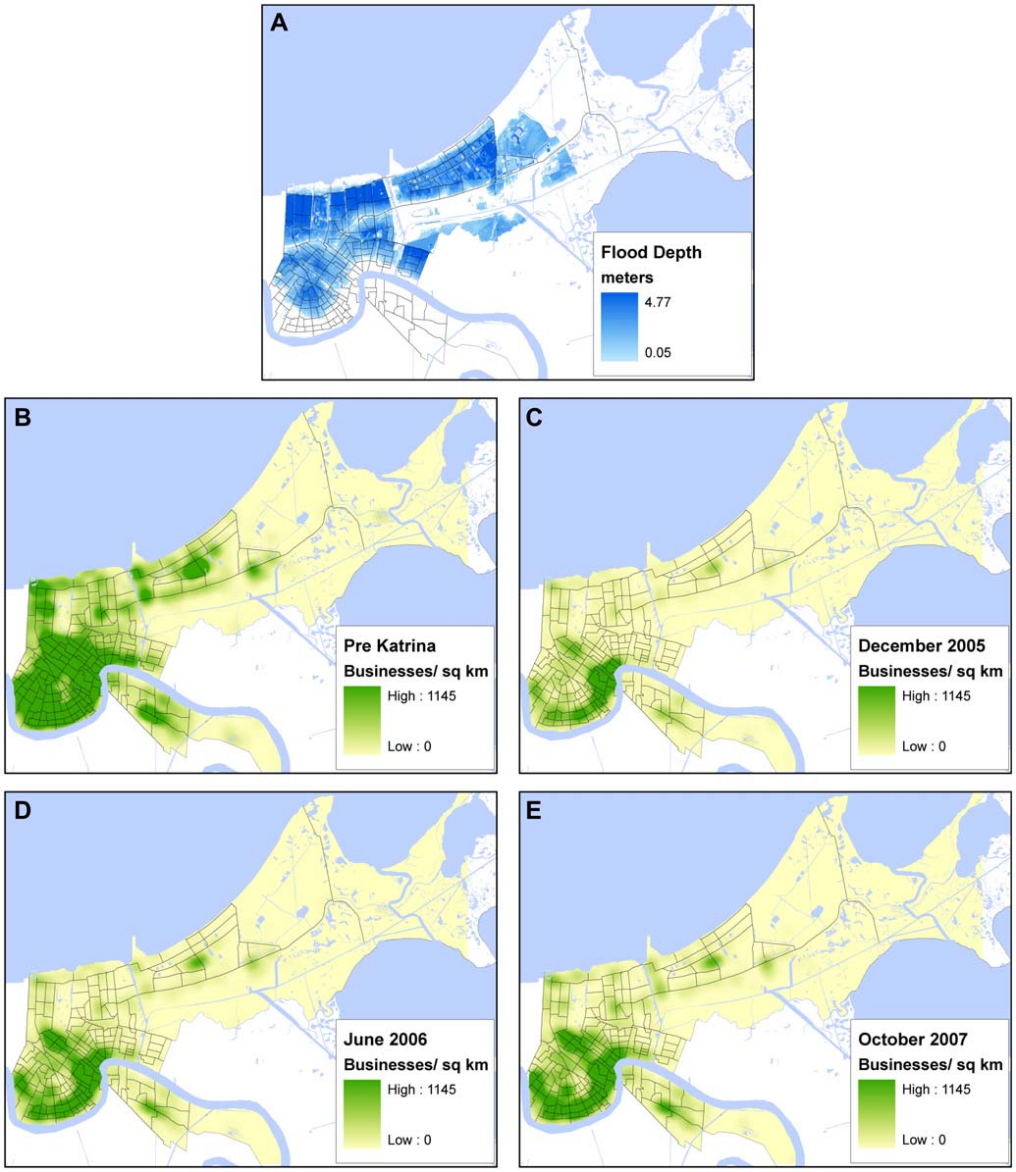
Kernel density maps of businesses reopened in the three time periods. The flood map shows flood depth as of September 2, 2005.

The discussion here focuses on a project to collect and analyze time-critical data on business return in New Orleans after Hurricane Katrina. We employed two types of data collection: telephone and street surveys. The telephone surveys involved all businesses in the Orleans Parish prior to Katrina and were timed to reflect a short term, intermediate and longer time interval, with the first (short term) taking place in December 2005, the second (intermediate term) in June 2006, and the third (longer term) in October 2007. The purpose of multiple-round surveys was to capture the spatial and temporal dynamics in factors viewed as important by businesses in their decision making, and enable subsequent quantitative modeling and prediction over both time and space. The street surveys were conducted weekly for three major commercial corridors for over three years, starting October 2005. The three commercial corridors chosen for the street survey included: St. Claude Avenue, Magazine Street, and Carrollton Avenue (Figure 1). St. Claude Avenue, which experienced light flooding after Katrina, traverses a struggling, working-class downtown neighborhood; Magazine Street serves middle- to upper-class uptown neighborhoods and suffered no flooding; Carrollton Avenue traverses both middle-class and working-class neighborhoods and suffered extreme flooding in some areas, some flooding in most areas, and none in others. These three corridors transect a wide range of socioeconomic, historical, and topographic conditions in the city, providing a useful means for comparison and validation with the results from the telephone survey.

We present analyses of business responses based on the three telephone surveys reflecting short, intermediate and longer term attitudes about factors that might influence decisions about re-opening after the hurricane. There was evidence of changing business views over the three different time intervals, and differences in responses when businesses were stratified according to their opening and flood statuses. Our survey results based on data collected over the two-year period following the disaster provide a rare but useful look at business attitudes that may influence decisions about re-opening in the aftermath of a disaster.

## Results

The time-series telephone survey results were analyzed to answer four major questions: [1] which types of businesses returned to operation most rapidly after the disaster and where were these located? In addition, we were interested in patterns of change in the types of businesses that re-opened over the intermediate and longer time horizons. [2] How did the survey responses differ between those businesses which remained close and those that were open at the various time horizons covered by our survey? In addition, we were interested in whether the responses of open and closed establishments changed over the various time horizons. [3] How did the survey responses differ by type of business, and was there variation in responses by different types of businesses over time? [4] Finally, how did the survey responses differ between those businesses that were flooded and those that were not, and did the responses change over time?

### Re-opening rates by business type

The survey response statistics (Table 1) reveal a grim picture of business openings in New Orleans in the first two years after Katrina. If we [1] assume that the “no answer” category (no answer after five or more contact attempts) and the “disconnected” category (indicating a disconnected phone) represent businesses that had not yet reopened, [2] assume that those who refused to participate in the surveys were businesses that were open, and [3] add in those who participated in the survey but indicated they were not open, then we find that less than 26% businesses opened in the entire Orleans Parish (City of New Orleans) in the first four months after Katrina. This business re-opening rate increased to 39% around 10 months after Katrina, and 66% two years after Katrina.

**Table 1:** Survey statistics of the three surveys.

	Dec05	Jun06	Oct07
Total sample	9132	9139	6155
Total attempted	8574	8808	5837
Others	359	439	2294
Revised (Attempted-Others)	8215	8369	3543
Completed survey	975(12%)	1418(17%)	1232(35%)
Assumed open	1173(14%)	1867(22%)	1101(31%)
Disconnected	1259 (15%)	2376(28%)	170(5%)
No answer	4808(59%)	2708(32%)	1040(29%)

Note: The percentage values were computed using “Revised” (Total attempted minus Others) as denominator. (Note: “Others” include “no eligible respondent”, “incorrect business”, and “not a business.”; whereas “Assumed open” include “hard or soft refusal”, “busy”, “call back”, “fax”, “already taken survey”, “partially completed”, and “mail back”.)

If we interpret the “disconnected” category as indicating short-term business decisions to not re-open, and the “no answer” category as reflecting businesses who were uncertain and taking a wait-and-see approach to the re-opening decision, then during the first four months after Katrina, 15% of the establishments made short-run decisions not to re-open anytime soon, and another 59% of businesses were in the uncertain (wait-and-see) category. The second survey reflected the intermediate term (around 10 months after the disaster) when uncertainty about the future was reduced. Here we find the percentage of “disconnected” increased to 28% while the percentage of “no answer” decreased to 32%. Of course, the latter percentage reflects a reduction in firms taking a wait-and-see approach in the intermediate term (which seems intuitively plausible), but also an increase in firms deciding not to re-open (anytime soon or never) from 15% to 28% at the 10 month point. At the two-year horizon after the disaster, the percentage of “disconnected” decreased substantially to 5%, whereas “no answer” remained relatively constant at 29%, indicating a stabilizing trend.

We classified business establishments in New Orleans into seven groups, based on their North American Industry Classification System (NAICS) codes which have been constructed to reflect similarity of economic function. Re-opening rates were calculated for establishments classified by these seven related groups (Tables 2 & 3). In the short-run, 33 percent of establishments that were open fell into Group 7 (professional, scientific and technical services), and 48% of open businesses in the intermediate term (10 months after the disaster) were of this type. In contrast, the average opening rates for all firms were 25% and 38% in the first two surveys. Group 4 (educational, health care, social assistance, and public administration) suffered the most in the first four months, with a reopening rate of only 17%. However, as time progressed, the difference in reopening rates among businesses classified into the seven groups diminished, with each of the groups approaching the average reopening rate of 65% for all businesses by October 2007, approximately twenty-six months after Katrina.

**Table 2:** The seven business groups and their North American Industry Classification System (NAICS) codes used in this paper.

Group	NAICS code	Description
1	11,21,22,23,31,32,33	Mining, utilities, construction, manufacturing, agriculture, forestry, fishing, and hunting
2	42,44,45	Wholesale and retail
3	51,52,53	Information, finance, insurance, and real estate
4	61,62,92	Educational, health care, social assistance, and public administration
5	71,72	Arts, entertainment, recreation, accommodation, and food services
6	48,49,55,56,81	Management of companies, waste management, transportation, warehousing, and other services
7	54	Professional, scientific, and technical services

**Table 3:** Business opening ratio by type at the three time periods.

Group	Dec05	Dec05	Dec05	Jun06	Jun06	Jun06	Oct07	Oct07	Oct07
	Total	Open	%Open	Total	Open	%Open	Total	Open	%0pen
1	601	128	21	627	234	37	279	178	64
2	1533	368	24	1589	593	37	681	464	68
3	880	233	26	882	361	41	367	237	65
4	975	166	17	1007	322	32	414	252	61
5	1034	266	26	1072	397	37	481	305	63
6	1750	390	22	1746	601	34	681	429	63
7	1401	464	33	1423	676	48	598	397	66
Sum/Ave	8174	2015	**25**	8346	3184	**38**	3501	2262	**65**

Note: The “Total” column is the sum of “Completed survey”, “Disconnect”, “No answer”, and “Assumed open” in Table 1. The “% open” figures were derived by assuming “Disconnect” and “No answer” as businesses closed. Note that within the “Completed survey” category, a small portion of businesses remained closed even though they participated in the survey.

We linked business openings with their geographic locations through the use of geographic information system (GIS) methods, and computed the business opening rate as a percentage of all businesses in each census tract for all the 181 tracts in Orleans Parish. For visualization purposes, the results were mapped using a kernel density smoothing method instead of the conventional tract-level choropleth map. This allows a comparison that emphasizes the uneven business density in the study area before and after Katrina. A map showing flood depths immediately after Katrina (September 2, 2005) is also shown (Figure 1). In the first (short-term) survey the majority of tracts (56% or 100 of the 181 tracts) had less than 20% of businesses open, and as one would expect, tracts having a larger proportion of open businesses were located in areas that had not been severely flooded. In the intermediate term (ten months after Katrina in June 2006), the number of census tracts with less than 20% businesses re-opened decreased from 56% to 28% (representing 50 tracts). These tracts with low re-opening rates were mostly located in areas that had higher flood depth in the eastern part of Orleans Parish (lower Ninth Ward), the mid-city, and Gentilly areas. Twenty-six months after (October 2007), there were still a few census tracts (4% or 7 tracts) scattered throughout the mid-city area where less than 20% businesses had reopened. Most census tracts exhibited reopening rates of 40% or above by this time.

### Overall survey responses

**Figure 2:**
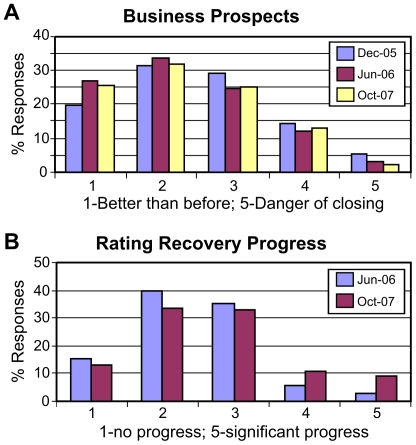
Business prospects and recovery progress rated by business owners in different time periods.

When businesses were asked to rate current prospects for their businesses, about 51% answered that their business prospects were better or about the same (rank 1 and 2) in the first survey, compared with 61% and 60% in the second and third surveys, respectively (Figure 2). Similarly, in the first survey about 19% answered that their businesses were struggling or in danger of closing (rank 4 and 5), compared with 15% and 14% in the second and third surveys. These results show a significant improvement in current business prospects between December 2005 and June 2006 (the first and second surveys), with an average rank score decreasing from 2.54 to 2.31 over the intermediate term. However, current business prospects remained virtually the same between the second and third surveys (rank scores of 2.31 and 2.30; see Table 4; also see Supplementary Tables S1, S2, S3).

**Table 4:** Average ranks of barriers and prospect for all businesses surveyed.

Problem	Dec-05	Jun-06	Oct-07
Damage	2.64	3.02	2.41
Insurance	2.66	2.64	2.58
Employee	2.73	2.97	2.69
Customer	2.89	2.76	2.68
Crime	–	2.41	***2.94***
Levee	***3.19***	***3.20***	2.87
Utilities	2.58	***3.15***	2.33
Communication	2.72	***3.18***	1.99
Environmental	2.23	2.42	1.82
Governmental	2.66	2.47	2.34
Financing	2.47	2.31	2.41
Prospect	2.54	2.31	2.30
Recovery Progress	–	2.41	2.68

Note: Except for the “recovery progress” variable, scores range from 1 to 5 and the higher the score, the more important the problem.

When businesses were asked to rate the economic recovery progress in Orleans Parish, about 19% businesses rated the recovery progress as satisfactory (rank 4 and 5) in the third survey, compared with only 9% in the second survey (Figure 2). (The question was not included in the first survey.) At the same time, it can also be said that about 56% businesses rated the recovery progress as unsatisfactory (rank 1 and 2) in June 2006, compared with 45% in October 2007. The average scores for the recovery progress improved from 2.41 to 2.68 between June 2006 and October 2007 (Table 4).

When business owners were asked to rate a series of problems considered barriers to return in a post-Katrina environment, the main concern was levee protection in the first survey, with an average score of 3.19, with the next highest concern being a lack of customers at 2.89 (Table 4). At the time of the second survey in June 2006 levees were still the greatest concern with an average score of 3.20, but business owners considered utilities and communications (mean rank values of 3.15 and 3.18, respectively) as two equally important issues they were facing. These were followed closely by two more barriers: damage to business premises (3.02) and a lack of employees (2.97). Furthermore, in the second survey, we inserted the variable “crime” as a potential barrier, but business owners did not indicate this was an important issue, reflected by its low average rank score of 2.41. In the third survey, crime became the top concern, reflected in the average score of (2.94) that slightly surpassed concerns regarding levee protection (2.87).

These results show that immediately after Katrina, levee protection was considered the most important problem by business owners. As the city recovered during the ten months after Katrina, levees were still the major concern, but utilities and communications joined this as equally important concerns. Twenty-six months after Katrina, as utilities and communications were re-established and work on levee protection was underway, business owners focus turned away from these larger infrastructure issues to those affecting their day-to-day operations. This is reflected in higher scores for items such as damage to business premises, lack of employees, and crime. Issues that were prominent in the second survey were no longer prominent in the third survey. These results coincide loosely with timing of an announcement by the U.S. Army Corps of Engineers to rebuild and strengthen the current levee system by June 2006.

The overall response results presented above were based on all businesses without consideration of the responses stratified by characteristics such as business type, status of the business as open or closed, and the depth of flooding affecting the establishments. A closer examination of survey responses reveals important differences when stratified by these variables. For example, in the second survey, although levee protection, utilities, and communications were identified as the most important barriers using average rank scores, responses exhibited a bi-modal distribution falling in the two extreme categories (ranks 1 and 5) [Supplementary Table S2]. In other words, about half of the businesses surveyed reported levee protection as the most important barrier, and half thought levee protection was the least important barrier. This bi-modal pattern also existed for barriers such as utilities, communications, damage to premises, and lack of employees. This dichotomy of responses reflects the complexity of factors that influence business return decisions. It also indicates that almost every business had major concerns that impacted decisions to re-open, but these concerns were not necessarily the same for all firms. To further explore this issue, we consider responses stratified by business subgroups.

### Rating barriers by business type

**Figure 3:**
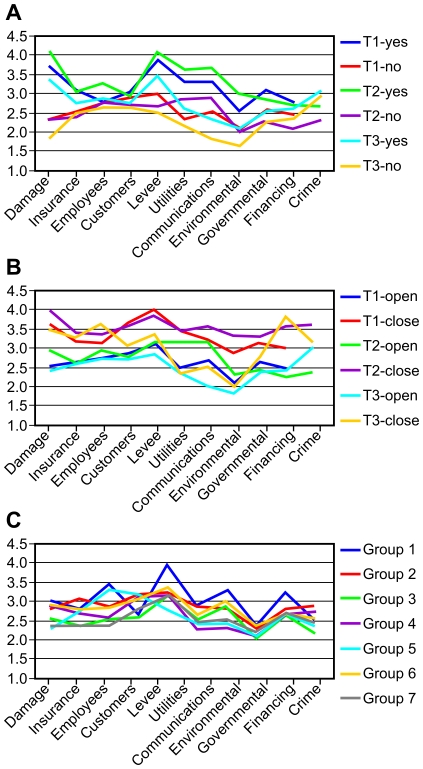
Rating of barriers by businesses according to their (A) flood status and (B) opening status in the three surveys, and (C) by business groups in survey-1.

A tabulation of the survey responses according to the seven business groups shows that Group 1, which included businesses in mining, utilities, construction, manufacturing, agriculture, forestry, fishing, and hunting, generally assigned higher barrier scores than other groups (Figure 3C). Levee protection was the top concern for this group, followed by the lack of employees, communication, and governmental problems. Group 3, which included businesses in information, finance, insurance, and real estate (FIRE), generally assigned the lowest barrier scores of the seven groups. The biggest contrast is that levee protection was ranked the highest (3.92) by Group 1, but the lowest (2.77) by Group 5, which includes businesses in arts, entertainment, recreation, accommodation, and food services (Supplementary Table S4). In contrast, top barriers for Group 5 were a lack of employees and customers, while the latter was considered the lowest barrier for Group 1 businesses.

These results indicate a great deal of variation in survey responses within and between groups, with no discernable pattern. A formal statistical discriminant analysis was conducted to determine if the groups could be clearly distinguished using the 11 barrier variables. The results indicated that only 26% of all businesses could be correctly classified using the response data from the first survey. This suggests that barrier variables are not capable of distinguishing/discriminating between businesses classified into the seven groupings. In other words, the seven groups derived using the NAICS codes were not distinctive in terms of their responses/attitudes regarding potential barriers. The percentage of businesses correctly classified by discriminant analysis carried out using the second and third surveys were about the same, 22% and 24% respectively. Based on this, we can conclude that other factors such as business open/closed status or flooded/non-flooded status may be more important than the NAICS codes (purportedly reflecting similarity of economic function) in explaining the survey responses.

### Rating barriers by open/closed status

When the survey responses were tabulated by the open/closed status of businesses, those businesses closed at the time of the survey gave consistently higher barrier scores than those that were open, as we might expect (Figure 3B; Supplementary Table S5). The barriers were perceived as even more important by owners of businesses still closed during the second survey, as indicated by survey response scores that were consistently the highest across all barrier variables. The top concern for businesses that remained closed shifted from levee protection in the first survey to damage to premises in the second survey. In the third survey, ratings of barriers by the businesses that remained closed were not consistent across the barrier variables. Financing became the top concern, followed by the lack of employees and levee protection.

A discriminant analysis that used the open/closed binary variable of each business as the group variable and the 11 barrier variables as predictors was conducted. The results show that about 70% of all the businesses were correctly classified into open or closed based on their survey responses in the first survey, and the classification accuracy slightly increased to 72% in both the second and third surveys. Based on the structural matrices derived from the discriminant analysis, the most important variable that distinguished between businesses that were opened versus businesses that remained closed was damage to premises in the first time period. In the second and third time periods, financing became the top variable that distinguished those businesses that were open from those that were not.

From an economic theory perspective, the difference in responses between open and closed businesses makes sense. For an open business, there is no marginal (additional) cost to open and so their concerns are with customers, employees, and other typical business challenges. If the business is profitable, it may pay to stay open even if another catastrophe could occur at any time. The potential for a catastrophe reduces the time horizon and thus the value of the business whether it is held by the existing owners or sold to new owners. Therefore, for a currently opened business long-term problems such as the potential damage from a natural disaster reduces the value of the business, the amount of optimal investment in the business, and the potential long-term market for their goods and services. For a closed business, the marginal cost of opening may be substantial and there has to be enough long-term benefit to support the substantial costs of re-opening. Therefore, a closed business must pay more attention to long-term prospects than an open business. Nonetheless, both types of firms profit as the long-term outlook improves, although the contrast is greatest for the closed firms.

### Rating barriers by flood status

Since the initial survey did not include a question regarding flood status, we derived this information by linking business locations with the LIDAR image data to determine whether each business in the survey was flooded or not. In the second and third surveys, a flood status question was included. When the ratings of barriers were tabulated by the flood status of businesses, similar results to that of the open/closed status were obtained. Businesses that were flooded gave higher scores than those that were not flooded. The barriers were perceived to be more serious in the second time period, as businesses that had been flooded gave the highest scores for almost all barrier variables (Figure 3A; Supplementary Table S6), with damage to premises ranked the highest, followed by levee protection and communications.

Results from the discriminant analysis conducted using the binary flood status as the group variable resulted in a 73% correct classification based on the barrier responses from the first survey. The classification accuracy increased to 79% and 74%, respectively, in the second and third surveys. The most important variables that distinguished those businesses flooded from those not flooded were very consistent for the three time periods. These were: damage to premises, followed by utilities in the second survey and levee protection in the second and third surveys.

The earlier comments regarding the economics of open/closed businesses are relevant here as well, since flooding impacts the chances that a business is open/closed as well as the marginal cost of opening for a closed business. In the absence of flooding, there is no marginal (additional) cost to open and so concerns of non-flooded establishments center on customers, employees, and other typical business challenges. For a flooded (or closed) business, the marginal cost of opening may be substantial and there has to be enough long-term benefit to support the substantial costs of re-opening. Therefore, a closed/flooded business must pay more attention to long-term prospects 'than an open/non-flooded business.

## Discussion

Literature directly addressing business return after a major natural disaster is relatively limited. Moreover, the findings from the already scanty literature are mixed and sometimes contradictory [13], [14]. Some studies argue that disasters have few effects beyond the immediate or short-term recovery periods [11], [12], while others conclude that at most, natural disasters exacerbate existing trends [15], [16]. Yet another group of studies actually suggests that climate-related disasters have long-term positive economic consequences related to physical capital, human capital, and productivity [17], [18]. Furthermore, most studies have been conducted at a regional scale, which may obscure heterogeneity in impacts of disasters on local communities. Conclusions about the economic impacts of a natural disaster made at the broad regional scale may be very different from those derived at finer spatial scales.

At a broader scale, earlier studies suggested that business decisions to locate or relocate could include factors such as future hurricane risks, economic impacts, insurance rates, emergency support, and market potential [10], [19]. At a local (fine spatial) scale, the literature generally shows that small businesses and non-profit organizations do not recover from major disasters [14], citing the ability, or rather the inability, to adapt as a crucial variable for these small businesses to recover [20]. For example, a study of the Northridge earthquake found that small businesses were more vulnerable and less likely to recover [9]. Webb and others compared business recovery patterns in Santa Cruz County, California after the Loma Prieta earthquake and South Dade County, Florida after Hurricane Andrew using surveys [11]. They found that most businesses did not experience long-term declines, and that retail businesses were less likely to recover after a natural disaster. However, they also acknowledged their study might be biased because surveys were conducted six and eight years after the disaster events. As noted earlier, “survival bias” can arise in these circumstances where surveys do not include businesses that closed in the short-term and/or those leaving the region. Based on Webb and others' findings, Waugh and Smith inferred that New Orleans would have a more difficult time recovering from Katrina due to its large tourism industry that consists of many small retail businesses [14]. Kates and others also pointed to a long reconstruction period for New Orleans noting the declining population trend of the city [15].

There is also a body of literature on vulnerability and resilience which could be used to shed light on our study of business decisions to re-open after a major disaster. However, the existing literature has seldom addressed how individual businesses aggregate to create a community [21]–[24]. Webb and others suggested the need for a multi-level conceptualization of long-term business vulnerability and resilience following disasters, which would include not only firm-level characteristics, but also neighborhood characteristics and the broader natural and socioeconomic environments [11], [12]. More recently, Zhang and others proposed a conceptual model of disaster impacts on businesses and identified a number of key factors that would increase or decrease business' vulnerability [16]. Although their models were not fully developed, their work highlighted the need for public policy research to help reduce business vulnerability to natural disasters.

For the most part, our findings support findings reported in past literature. Similar to previous studies, businesses in the professional, scientific and technical services were found to open more rapidly in the aftermath of Katrina, whereas businesses in educational, health care, social assistance, and public administration suffered most during the immediate aftermath (first four months). However, the differences in rates of business return among the seven different grouping of businesses by type became indistinguishable at a two year horizon after the disaster, with the average reopening rate for each of the groups approaching 65% by October 2007. This equaled the overall average rate of return for all businesses at the two-year horizon. We also note that the overall rate of business return was remarkably similar to the 67% rate of return for jobs reported by the Brookings Institute during the second quarter of 2007[25].

Unlike previous studies, we were able to include responses from individual businesses that remained closed at the time of each survey for a sequence of three surveys reflecting short-, intermediate- and long-term horizons for the aftermath of the Katrina disaster. The responses from businesses open and closed were significantly different, as revealed by a statistical discriminant analysis. Both groups of firms identified levee protection as the prime concern in the short-term, but diverged regarding which factors represented the greatest barriers to business return in the intermediate and longer-term horizons. For example, damage to business premises and financing problems were viewed as major obstacles by businesses that remained closed, whereas utilities, communications infrastructure and crime were major concerns of businesses that were open. Similar differences in responses were found for businesses that were flooded versus those that were not.

From an economic theory perspective, the difference in responses between open and closed (as well as flooded and non-flooded) businesses makes sense. As elaborated earlier, for an open business, there is no marginal (additional) cost to open and so their concerns are with customers, employees, and other typical business challenges. For a closed business, the marginal cost of opening may be substantial and there has to be enough long-term benefit to support the substantial costs or reopening. Therefore, a closed business must pay more attention to long-term prospects than an open business. Nonetheless, both types of firms profit as the long-term outlook improves, although the contrast is greatest for the closed firms.

In addition, open and closed businesses differ by their inherent vulnerability to disasters. If a business closes due to flooding, in the absence of more infrastructure the flooding will likely reoccur. In contrast, a business that survives a large natural disaster has excellent prospects of surviving future disasters of the same sort. Therefore, the priorities of these types of businesses will likely differ. Consequently, studies of only open or closed businesses suffer from sample selection bias where results from an analysis of one group may not apply to another group. Also, analyzing open and closed businesses together (without separate treatment) results in aggregation bias where the results based treating two groups as one are not truly representative of either group.

Multiple rounds of surveys conducted in this study provide a rare but revealing picture of how businesses attitudes change over time in an uncertain environment, and results of this type have not been reported in past literature. Three conclusions/insights can be highlighted. First, critical infrastructure protection of the impacted area, in this case levee protection, utilities, and telecommunication, stood out as prime concerns influencing business' decisions to return. This is expected and easily understood given the extreme levels of damaged caused by the Hurricane Katrina-induced levee failures.

Second, the associated impacts or collateral damages due to the disastrous event could play an even more significant role than the event itself in business' decisions to reopen. The large number of businesses (59%) that remained uncertain four months after Katrina indicates that business owners were weighting their options and taking a wait-and-see attitude. Businesses will likely return or re-open if there are timely and adequate recovery plans that can help repair property flooded or otherwise damaged by the event. Likewise, an emergency plan designed for rapid restoration of clean water, power, roads, public transportation, and telecommunications would help eliminate a major concern of businesses and presumably increase the likelihood of business return even in cases where establishments were not flooded. It is noteworthy that ten months after Katrina, businesses that were re-opened still considered levee protection, utilities, and communications as primary concerns, rather than more typical business concerns such as a lack of customers and employees. This points to a need for an adequate plan to protect the infrastructure in locations such as New Orleans that are subject to frequent hurricanes, since this would help minimize at-large impacts on the community that discourage the return of businesses.

These two conclusions underscore the importance of developing effective public policy and emergency plans to reduce business vulnerability and boost economic recovery. The results also point to the need to apply such policy in a timely manner before business decisions to return or close are made. Insofar as many of the disasters such as hurricanes or earthquakes associated with a particular area are well-known, as much as possible basic land use policies, building codes, and other regulations should be in place prior to the actual disaster. This reduces uncertainty on the part of businesses and consumers and will thus aid recovery and rebuilding. Moreover, large scale disasters usually result in governmental aid. However, the timing and amount of this aid is uncertain immediately after a disaster. In contrast, insurance programs, private or public, provide aid that automatically reflects the scope of the disaster. Encouraging individuals or businesses into insurance programs helps fund reserves for disaster recovery, provides more immediate aid, and helps maintain the locational value of vulnerable sites. The insurance program rules, if designed adequately, can promote resiliant rebuilding.

Third, an unexpected negative impact not documented in previous literature was increased crime in New Orleans after Katrina. A question regarding crime was included in the second survey, but businesses did not consider this an important issue in the intermediate term, ranking this with the lowest score. However, in the longer-term (twenty-six months after Katrina), crime rose to become the major concern of businesses, surpassing physical infrastructure and economic issues. The massive evacuation and displacement of families might have contributed to the breakdown of existing social networks in New Orleans ultimately leading to a dramatic increase in crime. It is clear that crime prevention (along with education and health services) can be considered an element of social infrastructure or adaptation policy that should improve societal resilience, which in turn increase business resilience and economic recovery.

Hurricane Katrina remains to be the costliest natural disaster to hit the United States both in terms of the number of people killed and property loss. There are many lessons to learn, and one frequently mentioned theme is the need to develop measures to prevent and mitigate the impacts. The results from the three surveys of businesses in the Orleans Parish are not completely new, but they provide empirical observations about business attitudes regarding infrastructure protection and other social adaptation policies needed to minimize spillover impacts and increase resilience and economic recovery. A major implication of the findings, which should be applicable to other major disasters in other localities, is that policies and regulations related to disaster prevention and mitigation should already be in place prior to the actual disaster to minimize uncertainty faced by business owners. Finally, we note that detailed planning strategies at a local level will need to rely on results based on detailed quantitative models, which are currently being developed based on the survey observations. Critical questions such as what factors influence individual business decisions to reopen, how re-opening of a business in one location would affect the decision of neighboring establishments to re-open (i.e., the spatial spillover impacts) and what types of aid strategies would be most effective will be addressed in the next phase of the project that focuses on spatial modeling.

## Materials and Methods

The telephone surveys were conducted for all businesses in the Orleans Parish in three different times: December 2005, June 2006, and October 2007. The purpose of multiple round surveys is to capture the spatial and temporal dynamics in business attitudes that influence their decision making and to enable subsequent quantitative modeling and prediction over both time and space. The first survey, conducted four months after Katrina, provided the first business outlook of New Orleans after Katrina, thus the preliminary report of its results generated wide national and local media attention. The second survey, conducted ten months after Katrina, followed the same survey instrument with minor modifications, so that the results can be used to track the changing patterns of business return through time after a major catastrophe. The third survey, conducted 26 months after Katrina, was in collaboration with the Louisiana Recovery Authority.

All three surveys were conducted for the entire Orleans Parish (county) using the August 2005 Louisiana Department of Labor Micro File for Economic Development in the greater New Orleans Area, which includes also Jefferson and St. Tammany Parishes. There were about 33,000 businesses in all three parishes that existed before Katrina. The file contains confidential information of about 45 variables for each business establishment. Variables that are of especially useful for our research include: the NAICS code (National Association of Industry Code) classification of the type of business, names of the businesses, physical address, telephone, contact person, longitude and latitude, zip code, census tract, census block, parish location, number of employees, and aggregate wages. The surveys were conducted with assistance from the Louisiana State University Public Policy Research Laboratory.

As in any typically large data file, there are coding mistakes and missing data. A number of tedious steps were taken to correct and validate the business establishment file before the phone survey. For example, some of the longitude and latitude coordinates for the location of businesses did not match the parish or zip code locations. We utilized GIS methods such as address matching and mapping to correct all the errors we identified. We then extracted only the records for Orleans Parish, which resulted in a total of about 11,000 businesses before Katrina. Special attention was paid to multi-establishment firms which had several locations (e.g., national chains such as Wal-Mart and fast food franchises), thus resulting in several records in the file. These firms listed a single contact person, allowing us to extract “unique” records to avoid duplication. This resulted in about 10,000 unique businesses in Orleans Parish that were used for the phone survey.

The survey questions were designed to be short and direct to maximize participation. Also, questions were kept basically the same for all three surveys to enable comparison over time. However, since we combined our third survey with the research group from the Louisiana Recovery Authority, the third survey was much longer than the previous two, but the first part of the questions is the same as the previous two surveys to enable comparison. The three main questions asked in most of the three surveys were: [1] Are you open for business? If not, when do you plan to open? Was your business flooded? [2] Rank the major problems/barriers for your businesses from 1 to 5, with 1 being no problem at all and 5 being a major problem: damage to Premises? Insurance? Lack of employees? Lack of customer base? levee protection? Suppliers? Utilities? Communications? Environmental problems? Governmental problems? Financing? Crime? Others?) [3] How optimistic are you about the future of your business? In addition, we had an open-ended question for businesses to identify the most effective ways to help economic recovery.

In Table 1, a business was put into the “No answer” category if there were no answer after five or more attempts at contact, which is interpreted as businesses that had not yet reopened. The “Disconnected” category represents a disconnected phone line, which leads to the inference that the business never reopened. Of those who answered calls, some completed the survey and these responses were designated as “Complete,” whereas the “Others” category included: hard or soft refusal to complete the survey, no eligible respondents, surveys to be faxed or mailed, partial completion, and other data file errors. Together, the “Completed” and “Others” categories are interpreted as businesses that are open or have the potential to open at the time of the survey.

The flood map in Figure 1 was created from a raster file representing the flood depth by September 02, 2005. The file was originated by the U.S. Army corps of Engineers and can be accessed from Louisiana State University GIS Information Clearinghouse, CADGIS Research Laboratory, via the website: http://www.katrina.lsu.edu. The dataset, defined in ArcGIS grid file format with a spatial resolution of 25 meters, covers Orleans, Jefferson and St. Bernard parishes.

The kernel density maps were created using a spatial resolution of 100 meters and a search radius (kernel size) of 1000 meters via the software ArcGIS. A larger kernel size would smooth the result, while a smaller one will have the opposite effect. The first and second surveys comprise in practical terms the whole population of the study area (all businesses in Orleans Parish), while the third survey was constrained with the resources and collaboration with the Louisiana Recovery Authority, resulting in only a sample of businesses surveyed, even though it is a large sample. This creates problem for mapping the business density from the third survey. In order to visually compare the results of the kernel maps of the three surveys, we added to the analysis a weight factor. In the pre-Katrina case, the weight value was set to 1 for all the businesses. In the first and second surveys, the weight factor was set to 1 for businesses that were opened and 0 for businesses that were closed. For the third survey, the weight factor for opened businesses was set as the ratio between the total number of businesses attempted in the second survey and that of the third survey (8346/3501 = 2.38) (see Table 3).

### Ethics Statement

This study has been exempted from institutional oversight by the Louisiana State University Institutional Review Board (Exemption number E3552).

## Supporting Information

Table S1 Summary of attributes of the first survey (December 2005) in frequency count and percentage (in brackets).
(0.04 MB DOC)

Table S2 Summary of attributes of the second survey (June 2006) in frequency count and percentage (in brackets).
(0.05 MB DOC)

Table S3 Summary of attributes of the third survey (October 2007) in frequency count and percentage (in brackets).
(0.04 MB DOC)

Table S4 Average ratings of barriers tabulated by business group in the first survey (N is the number of businesses in each group after excluding missing value in at least one variable).
(0.04 MB DOC)

Table S5 Average ratings of barriers tabulated by business' opening status in the three surveys.
(0.03 MB DOC)

Table S6 Average ratings of barriers by business' flood status in the three surveys.
(0.03 MB DOC)
