# Supplementary material for: Business Return in New Orleans: Decision Making Amid Post-Katrina Uncertainty
Source: PLoS One. 2009 Aug 26;4(8):e6765. doi: 10.1371/journal.pone.0006765 (PMC2727799; doi:10.1371/journal.pone.0006765)
Supplement: Table S1 — Summary of attributes of the first survey (December 2005) in frequency count and percentage (in brackets). (0.04 MB DOC) [file pone.0006765.s001.doc]

|  | AVG | STD | -9-0 | 1 | 2 | 3 | 4 | 5 |
| --- | --- | --- | --- | --- | --- | --- | --- | --- |
| Open? | 1.10 | 0.30 | 1 | 844(90) | 92(10) | 0 | 0 | 0 |
| Damage | 2.64 | 1.58 | 3 | 339(36) | 156(17) | 148(16) | 80(9) | 211(23) |
| Insurance | 2.66 | 1.63 | 129 | 326(40) | 81(10) | 134(17) | 74(9) | 193(24) |
| Employee | 2.73 | 1.61 | 2 | 353(38) | 86(9) | 171(18) | 108(12) | 217(23) |
| Customer | 2.89 | 1.54 | 13 | 272(29) | 118(13) | 191(21) | 126(14) | 217(24) |
| Supplier | 2.24 | 1.34 | 30 | 394(43) | 162(18) | 178(20) | 88(10) | 85(9) |
| Levee | 3.19 | 1.82 | 42 | 312(35) | 64(7) | 64(7) | 51(6) | 404(45) |
| Utilities | 2.58 | 1.58 | 17 | 379(41) | 107(12) | 141(15) | 109(12) | 184(20) |
| Communication | 2.72 | 1.55 | 7 | 319(34) | 130(14) | 166(18) | 119(13) | 196(21) |
| Environmental | 2.23 | 1.42 | 24 | 438(41) | 129(14) | 155(17) | 83(9) | 108(12) |
| Governmental | 2.66 | 1.60 | 42 | 348(39) | 105(12) | 144(16) | 100(11) | 198(22) |
| Financing | 2.47 | 1.55 | 63 | 385(44) | 97(11) | 144(17) | 94(11) | 154(18) |
| Prospect | 2.54 | 1.12 | 33 | 178(20) | 284(31) | 265(29) | 128(14) | 49(5) |

Note: Total number of samples: 937. For all questions, the higher the average values, the more serious the issue. For Supplementary Tables 1-3, averages and standard deviations (STD) were computed after excluding missing values. The category of -9-0 indicates missing values which include -8: Do not know; -9: Refuse to answer, and other invalid values.
