# Supplementary material for: Business Return in New Orleans: Decision Making Amid Post-Katrina Uncertainty
Source: PLoS One. 2009 Aug 26;4(8):e6765. doi: 10.1371/journal.pone.0006765 (PMC2727799; doi:10.1371/journal.pone.0006765)
Supplement: Table S2 — Summary of attributes of the second survey (June 2006) in frequency count and percentage (in brackets). (0.05 MB DOC) [file pone.0006765.s002.doc]

|  | AVG | STD | -9-0 | 1 | 2 | 3 | 4 | 5 |
| --- | --- | --- | --- | --- | --- | --- | --- | --- |
| Open? | 1.06 | 0.23 | 1 | 1317(94) | 78(6) |  |  |  |
| Flooded | 1.62 | 0.49 | 2 | 535(38) | 859(62) |  |  |  |
| Damage | 3.02 | 1.59 | 11 | 374(27) | 198(14) | 244(18) | 162(12) | 407(29) |
| Insurance | 2.64 | 1.62 | 155 | 513(41) | 105(9) | 225(18) | 117(9) | 281(23) |
| Employee | 2.97 | 1.65 | 16 | 456(33) | 131(10) | 205(15) | 180(13) | 408(30) |
| Customer | 2.76 | 1.60 | 19 | 489(36) | 161(12) | 256(19) | 133(10) | 338(25) |
| Crime | 2.41 | 1.59 | 21 | 656(48) | 152(11) | 172(13) | 134(10) | 261(19) |
| Levee | 3.20 | 1.76 | 58 | 440(33) | 86(6) | 138(10) | 108(8) | 566(42) |
| Utilities | 3.15 | 1.64 | 26 | 373(27) | 150(11) | 223(16) | 150(11) | 474(35) |
| Communication | 3.18 | 1.65 | 15 | 375(27) | 153(11) | 199(14) | 150(11) | 504(37) |
| Environmental | 2.42 | 1.58 | 24 | 644(47) | 158(12) | 186(14) | 124(9) | 260(19) |
| Governmental | 2.47 | 1.58 | 71 | 593(45) | 150(11) | 205(16) | 120(9) | 257(19) |
| Financing | 2.31 | 1.53 | 90 | 648(50) | 142(11) | 184(14) | 125(10) | 207(16) |
| Prospect | 2.31 | 1.09 | 30 | 365(27) | 457(34) | 337(25) | 163(12) | 44(3) |
| Preparedness | 1.26 | 0.44 | 34 | 1002(74) | 360(26) |  |  |  |
| Recov. Progress | 2.41 | 0.94 | 22 | 216(16) | 548(40) | 483(35) | 81(6) | 46(3) |

Note: Total number of samples: 1396. For all issues except “Recovery Progress”, the higher the average values, the more serious the issues.
