# Supplementary material for: Business Return in New Orleans: Decision Making Amid Post-Katrina Uncertainty
Source: PLoS One. 2009 Aug 26;4(8):e6765. doi: 10.1371/journal.pone.0006765 (PMC2727799; doi:10.1371/journal.pone.0006765)
Supplement: Table S3 — Summary of attributes of the third survey (October 2007) in frequency count and percentage (in brackets). (0.04 MB DOC) [file pone.0006765.s003.doc]

|  | AVG | STD | -9-0 | 1 | 2 | 3 | 4 | 5 |
| --- | --- | --- | --- | --- | --- | --- | --- | --- |
| Open? | 1.02 | 0.15 | 0 | 1161(98) | 29(2) |  |  |  |
| Flooded | 1.61 | 0.49 | 5 | 466(39) | 719(61) |  |  |  |
| Damage | 2.41 | 1.61 | 13 | 555(47) | 161(14) | 132(11) | 80(7) | 249(21) |
| Insurance | 2.58 | 1.65 | 81 | 498(45) | 91(8) | 151(14) | 120(11) | 249(22) |
| Employee | 2.69 | 1.59 | 12 | 450(38) | 134(11) | 169(14) | 185(16) | 240(20) |
| Customer | 2.68 | 1.54 | 19 | 421(36) | 150(13) | 211(18) | 160(14) | 229(20) |
| Crime | 2.94 | 1.63 | 15 | 380(32) | 128(11) | 173(15) | 170(14) | 324(28) |
| Levee | 2.87 | 1.72 | 87 | 433(39) | 76(7) | 136(12) | 115(10) | 343(31) |
| Utilities | 2.33 | 1.48 | 23 | 550(47) | 134(11) | 188(16) | 141(12) | 154(13) |
| Communication | 1.99 | 1.36 | 16 | 678(58) | 141(12) | 142(12) | 111(9) | 102(9) |
| Environmental | 1.82 | 1.26 | 24 | 727(62) | 160(14) | 121(10) | 79(7) | 79(7) |
| Governmental | 2.34 | 1.58 | 38 | 578(50) | 124(11) | 140(12) | 99(9) | 211(18) |
| Financing | 2.41 | 1.56 | 40 | 531(46) | 137(12) | 149(13) | 140(12) | 193(17) |
| Prospect | 2.30 | 1.07 | 23 | 312(27) | 390(33) | 300(26) | 134(11) | 31(3) |
| Recov. Progress | 2.68 | 1.10 | 0 | 169(14) | 369(31) | 424(36) | 133(11) | 95(8) |

Note: Total number of samples: 1190. For all issues except “Recovery Progress”, the higher the average values, the more serious the issues.
