# Supplementary material for: Business Return in New Orleans: Decision Making Amid Post-Katrina Uncertainty
Source: PLoS One. 2009 Aug 26;4(8):e6765. doi: 10.1371/journal.pone.0006765 (PMC2727799; doi:10.1371/journal.pone.0006765)
Supplement: Table S4 — Average ratings of barriers tabulated by business group in the first survey (N is the number of businesses in each group after excluding missing value in at least one variable). (0.04 MB DOC) [file pone.0006765.s004.doc]

|  | Group1 | Group2 | Group3 | Group4 | Group5 | Group6 | Group7 |
| --- | --- | --- | --- | --- | --- | --- | --- |
| Damage | 3.02 | 2.78 | 2.55 | 2.86 | 2.27 | 2.89 | 2.33 |
| Insurance | 2.77 | 3.04 | 2.36 | 2.65 | 2.70 | 2.76 | 2.37 |
| Employees | 3.44 | 2.86 | 2.53 | 2.55 | 3.31 | 2.80 | 2.34 |
| customers | 2.65 | 3.14 | 2.57 | 3.09 | 3.15 | 3.02 | 2.81 |
| crime | -- | -- | 2.11 | 2.23 | 2.09 | 2.23 | 2.17 |
| levee | 3.92 | 3.20 | 3.13 | 3.12 | 2.77 | 3.36 | 3.15 |
| utilities | 2.90 | 2.86 | 2.55 | 2.25 | 2.41 | 2.64 | 2.43 |
| communications | 3.27 | 2.81 | 2.89 | 2.28 | 2.42 | 3.00 | 2.55 |
| environmental | 2.35 | 2.28 | 2.04 | 2.06 | 2.09 | 2.38 | 2.19 |
| governmental | 3.19 | 2.80 | 2.61 | 2.55 | 2.70 | 2.61 | 2.61 |
| financing | 2.46 | 2.86 | 2.16 | 2.71 | 2.37 | 2.55 | 2.43 |
| N | 52 | 133 | 97 | 69 | 91 | 122 | 166 |
