# Supplementary material for: Business Return in New Orleans: Decision Making Amid Post-Katrina Uncertainty
Source: PLoS One. 2009 Aug 26;4(8):e6765. doi: 10.1371/journal.pone.0006765 (PMC2727799; doi:10.1371/journal.pone.0006765)
Supplement: Table S5 — Average ratings of barriers tabulated by business' opening status in the three surveys. (0.03 MB DOC) [file pone.0006765.s005.doc]

|  | T1-open | T1-close | T2-open | T2-close | T3-open | T3-close |
| --- | --- | --- | --- | --- | --- | --- |
| Damage | 2.52 | 3.61 | 2.96 | 3.97 | 2.41 | 3.45 |
| Insurance | 2.59 | 3.17 | 2.61 | 3.36 | 2.57 | 3.25 |
| Employees | 2.72 | 3.11 | 2.94 | 3.34 | 2.70 | 3.60 |
| customers | 2.86 | 3.62 | 2.76 | 3.54 | 2.68 | 3.05 |
| crime | -- | -- | 2.38 | 3.59 | 3.00 | 3.15 |
| levee | 3.11 | 3.96 | 3.17 | 3.83 | 2.86 | 3.35 |
| utilities | 2.48 | 3.44 | 3.15 | 3.44 | 2.34 | 2.35 |
| communications | 2.67 | 3.23 | 3.19 | 3.53 | 2.02 | 2.50 |
| environmental | 2.13 | 2.85 | 2.36 | 3.31 | 1.83 | 2.00 |
| governmental | 2.65 | 3.11 | 2.46 | 3.29 | 2.36 | 2.75 |
| financing | 2.47 | 2.99 | 2.26 | 3.53 | 2.42 | 3.80 |
| N | 661 | 71 | 1070 | 70 | 972 | 20 |

Note: T1-December 2005, T2-June 2006, T3-October 2007; N is the number of businesses used in each category after excluding missing value in at least one variable.
