# Supplementary material for: Business Return in New Orleans: Decision Making Amid Post-Katrina Uncertainty
Source: PLoS One. 2009 Aug 26;4(8):e6765. doi: 10.1371/journal.pone.0006765 (PMC2727799; doi:10.1371/journal.pone.0006765)
Supplement: Table S6 — Average ratings of barriers by business' flood status in the three surveys. (0.03 MB DOC) [file pone.0006765.s006.doc]

|  | T1-yes | T1-no | T2-yes | T2-no | T3-yes | T3-no |
| --- | --- | --- | --- | --- | --- | --- |
| Damage | 3.73 | 2.30 | 4.14 | 2.32 | 3.39 | 1.86 |
| Insurance | 3.11 | 2.52 | 3.07 | 2.39 | 2.77 | 2.47 |
| Employees | 2.81 | 2.74 | 3.29 | 2.77 | 2.90 | 2.62 |
| customers | 3.02 | 2.91 | 2.95 | 2.72 | 2.75 | 2.65 |
| crime | -- | -- | 2.68 | 2.32 | 3.09 | 2.95 |
| levee | 3.87 | 2.99 | 4.07 | 2.68 | 3.46 | 2.52 |
| utilities | 3.32 | 2.36 | 3.64 | 2.87 | 2.61 | 2.17 |
| communications | 3.32 | 2.53 | 3.71 | 2.89 | 2.35 | 1.83 |
| environmental | 2.56 | 2.09 | 3.03 | 2.03 | 2.10 | 1.66 |
| governmental | 3.10 | 2.57 | 2.86 | 2.29 | 2.54 | 2.26 |
| financing | 2.79 | 2.44 | 2.72 | 2.10 | 2.62 | 2.35 |
| N | 167 | 565 | 438 | 702 | 370 | 618 |

Note: Yes for flooded, no for not flooded. T1-December 2005, T2-June 2006, T3-October 2007, N is the number of businesses in each category after excluding missing value in at least one variable.
